# Long-Acting Rilpivirine (RPV) Preexposure Prophylaxis Does Not Inhibit Vaginal Transmission of RPV-Resistant HIV-1 or Select for High-Frequency Drug Resistance in Humanized Mice

**DOI:** 10.1128/JVI.01912-19

**Published:** 2020-03-31

**Authors:** Kevin Melody, Chandra N. Roy, Christopher Kline, Mackenzie L. Cottrell, Dwayne Evans, Kathleen Shutt, Pleuni S. Pennings, Brandon F. Keele, Moses Bility, Angela D. M. Kashuba, Zandrea Ambrose

**Affiliations:** a Department of Infectious Diseases and Microbiology, Graduate School of Public Health, University of Pittsburgh, Pittsburgh, Pennsylvania, USA; b Department of Microbiology and Molecular Genetics, School of Medicine, University of Pittsburgh, Pittsburgh, Pennsylvania, USA; c Eshelman School of Pharmacy, University of North Carolina, Chapel Hill, North Carolina, USA; d Department of Biology, San Francisco State University, San Francisco, California, USA; e Division of Infectious Diseases, Department of Medicine, School of Medicine, University of Pittsburgh, Pittsburgh, Pennsylvania, USA; f AIDS and Cancer Virus Program, Frederick National Laboratory for Cancer Research, Frederick, Maryland, USA; Icahn School of Medicine at Mount Sinai

**Keywords:** HIV-1, NNRTI, PrEP, animal model, drug resistance, humanized mice, preexposure prophylaxis, rilpivirine, vaginal transmission

## Abstract

As a long-acting formulation of the nonnucleoside reverse transcriptase inhibitor rilpivirine (RPV LA) has been proposed for use as preexposure prophylaxis (PrEP) and the prevalence of transmitted RPV-resistant viruses can be relatively high, we evaluated the efficacy of RPV LA to inhibit vaginal transmission of RPV-resistant HIV-1 in humanized mice. Vaginal challenges of wild-type (WT), Y181C, and Y181V HIV-1 were performed in mice left untreated or after RPV PrEP. Plasma viremia was measured for 7 to 10 weeks, and single-genome sequencing was performed on plasma HIV-1 RNA in mice infected during PrEP. RPV LA significantly prevented vaginal transmission of WT HIV-1 and Y181C HIV-1, which is 3-fold resistant to RPV. However, it did not prevent transmission of Y181V HIV-1, which has 30-fold RPV resistance in the viruses used for this study. RPV LA did delay WT HIV-1 dissemination in infected animals until genital and plasma RPV concentrations waned. Animals that became infected despite RPV LA PrEP did not acquire new RPV-resistant mutations above frequencies in untreated mice or untreated people living with HIV-1, and the mutations detected conferred low-level resistance. These data suggest that high, sustained concentrations of RPV were required to inhibit vaginal transmission of HIV-1 with little or no resistance to RPV but could not inhibit virus with high resistance. HIV-1 did not develop high-level or high-frequency RPV resistance in the majority of mice infected after RPV LA treatment. However, the impact of low-frequency RPV resistance on virologic outcome during subsequent antiretroviral therapy still is unclear.

**IMPORTANCE** The antiretroviral drug rilpivirine was developed into a long-acting formulation (RPV LA) to improve adherence for preexposure prophylaxis (PrEP) to prevent HIV-1 transmission. A concern is that RPV LA will not inhibit transmission of drug-resistant HIV-1 and may select for drug-resistant virus. In female humanized mice, we found that RPV LA inhibited vaginal transmission of WT or 3-fold RPV-resistant HIV-1 but not virus with 30-fold RPV resistance. In animals that became infected despite RPV LA PrEP, WT HIV-1 dissemination was delayed until genital and plasma RPV concentrations waned. RPV resistance was detected at similar low frequencies in untreated and PrEP-treated mice that became infected. These results indicate the importance of maintaining RPV at a sustained threshold after virus exposure to prevent dissemination of HIV-1 after vaginal infection and low-frequency resistance mutations conferred low-level resistance, suggesting that RPV resistance is difficult to develop after HIV-1 infection during RPV LA PrEP.

## Abstract (precis)

The antiretroviral drug rilpivirine was developed into a long-acting formulation (RPV LA) to improve adherence for preexposure prophylaxis (PrEP) to prevent HIV-1 transmission. A concern is that RPV LA will not inhibit transmission of drug-resistant HIV-1 and may select for drug-resistant virus. In female humanized mice, we found that RPV LA inhibited vaginal transmission of WT or 3-fold RPV-resistant HIV-1 but not virus with 30-fold RPV resistance. In animals that became infected despite RPV LA PrEP, WT HIV-1 dissemination was delayed until genital and plasma RPV concentrations waned. RPV resistance was detected at similar low frequencies in untreated and PrEP-treated mice that became infected. These results indicate the importance of maintaining RPV at a sustained threshold after virus exposure to prevent dissemination of HIV-1 after vaginal infection and low-frequency resistance mutations conferred low-level resistance, suggesting that RPV resistance is difficult to develop after HIV-1 infection during RPV LA PrEP.

## INTRODUCTION

Approximately 1.7 million people became infected with human immunodeficiency virus type 1 (HIV-1) globally in 2018, with women aged 15 to 24 years accounting for 20% of these new infections (1). Daily, oral preexposure prophylaxis (PrEP) consisting of the nucleoside analogs tenofovir (TFV) disoproxil fumarate and emtricitabine (FTC), known together as Truvada, is currently recommended by the United States Centers for Disease Control and Prevention and the World Health Organization for individuals who are at high risk of acquiring HIV-1 infection. Drug concentrations that were detectable or above specific thresholds in the plasma (2–5) and rectal tissue (2) were associated with reduced HIV-1 transmission in Truvada PrEP clinical trials. However, adherence to the dosing regimen is one of the greatest challenges for PrEP efficacy (6).

The nonnucleoside reverse transcriptase inhibitor (NNRTI) rilpivirine (RPV) is FDA approved as a component of combination antiretroviral therapy (ART) that includes TFV disoproxil fumarate and FTC in a once-daily oral pill. RPV in combination with other antiretroviral drugs has been evaluated for efficacy as ART (7–9) and postexposure prophylaxis (10). RPV also has been developed in a nanoparticle long-acting formulation (RPV LA) to improve adherence (11, 12). Plasma mean peak concentrations (*C*_max_) after a 1,200-mg RPV LA dose were reported to be 140 to 160 ng/ml (13, 14), or approximately 12- to 13-fold higher than the reported protein-adjusted 90% effective concentration (EC_90_) of 12.1 ng/ml (15). Twenty-eight days after receiving the 1,200-mg dose, the mean plasma RPV concentrations were 63 to 83 ng/ml (13, 14). Clinical trials have evaluated RPV LA pharmacokinetics for PrEP (e.g., HPTN 076 and SSAT 040 studies) or combined with an integrase inhibitor for maintenance ART after virus suppression has been achieved (e.g., the LATTE-2 study) (16).

Only an estimated 38% of people living with HIV-1 (PLWH) are virally suppressed throughout the world (17). HIV-1 drug resistance has been shown to occur via preexisting mutations prior to therapy (18) and by selection during suboptimal therapy (19). Even drug-resistant mutations at frequencies below detection by population sequencing, including NNRTI-associated resistance mutations, can contribute to treatment failure in PLWH (20–23). Mutations that arise in NNRTI-containing regimens and that confer NNRTI resistance often lead to cross-resistance to multiple NNRTIs (24). For example, Y181C confers high-level resistance to nevirapine (>40-fold) but low-level resistance to etravirine and rilpivirine (3- to 4-fold), while Y181I/V confers >60-fold resistance to nevirapine and 12- to 15-fold resistance to etravirine and RPV (25). Y181C/I/V confers little resistance to efavirenz (EFV; 2- to 3-fold) (25).

The prevalence of RPV-resistant isolates ranges from 4 to 10% in individuals who are treatment naive (26–33) to 42 to 72% in individuals with viremia during non-RPV-containing ART (34–41). Three clinical studies evaluated the efficacy of RPV-containing regimens compared to EFV-containing regimens in treatment-naive individuals (the ECHO, THRIVE, and STaR trials), all of which identified multiple RPV-associated resistance mutations that arose in subjects on RPV-containing ART (42, 43). In the ECHO and THRIVE studies, Y181C/I was detected in 8% of individuals failing RPV-containing ART, particularly in subjects who had >100,000 baseline plasma viral RNA copies/ml (43). Additionally, in the SPIRIT study, which evaluated HIV-infected individuals switching from protease inhibitor-based ART to RPV/FTC/TDF who lacked preexisting NNRTI resistance mutations in plasma viral RNA, 4% of individuals acquired Y181C (2/51), with one experiencing treatment failure (44).

As RPV LA has been proposed for use as PrEP and the prevalence of transmitted RPV-resistant viruses can be 6% or higher in some populations (45, 46), we wanted to determine how effectively RPV LA PrEP could inhibit vaginal transmission of wild-type (WT) HIV-1 as well as two mutants that confer resistance to RPV in the humanized bone marrow, liver, and thymus (BLT) mouse model (47). We evaluated Y181C, which confers 3-fold resistance to RPV (25) and is one of the most frequently transmitted NNRTI-resistant mutations globally (48), and the less prevalent Y181V mutation (49), which was previously reported to confer approximately 20-fold resistance to RPV (50).

## RESULTS

### *In vitro* RPV susceptibility and replication of WT HIV-1_NL4-BAL_ and Y181C/I/V mutants.

**FIG 1:**
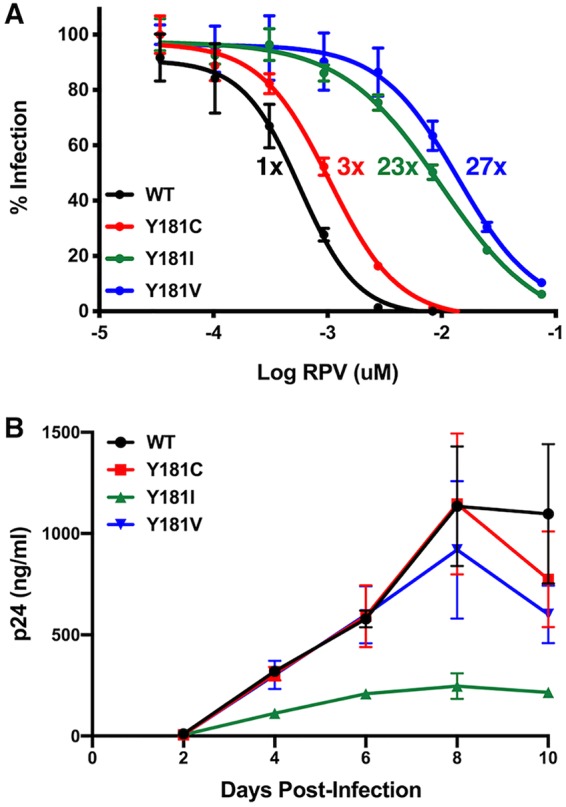
*In vitro* HIV-1_NL4-BAL_ susceptibility to RPV and replication. (A) RPV susceptibility of WT, Y181C, Y181I, and Y181V HIV-1_NL4-BAL_ was measured in TZM-bl cells in the presence of multiple concentrations of RPV. The results are representative of 3 independent experiments, each performed in triplicate. Error bars represent standard deviations (SD). Average fold changes in resistance for each of the mutants compared to that of WT HIV-1_NL4-BAL_ are designated next to each curve. (B) Replication of WT, Y181C, Y181I, and Y181V HIV-1_NL4-BAL_ was measured in human primary PBMCs from 3 individual donors by production of p24 in cell culture supernatants over time. Results are shown as means with standard errors of the means (SEM).

Mutations at amino acid 181 in HIV-1 reverse transcriptase (RT) have been shown to confer different levels of RPV resistance (25). RPV inhibition of WT HIV-1_NL4-BAL_, a CCR5-tropic, laboratory-adapted subtype B molecular clone, was compared to that of viruses with the mutations Y181C, Y181I, and Y181V in TZM-bl cells (Fig. 1A). The 50% effective concentration (EC_50_) of RPV for WT HIV-1_NL4-BAL_ was 0.54 ± 0.18 nM (0.20 ± 0.066 ng/ml), while Y181C, Y181I, and Y181V had 3-, 23-, and 27-fold increases in EC_50_, respectively. Replication of these viruses was tested in human peripheral blood mononuclear cells (PBMCs) isolated from HIV-negative donors (Fig. 1B). WT HIV-1_NL4-BAL_ replication in PBMC was similar to that of Y181C HIV-1_NL4-BAL_ and Y181V HIV-1_NL4-BAL_. While Y181I HIV-1_NL4-BAL_ had RPV susceptibility similar to that of Y181V HIV-1_NL4-BAL_, this virus replicated less well than the other viruses *in vitro*.

**FIG 2:**
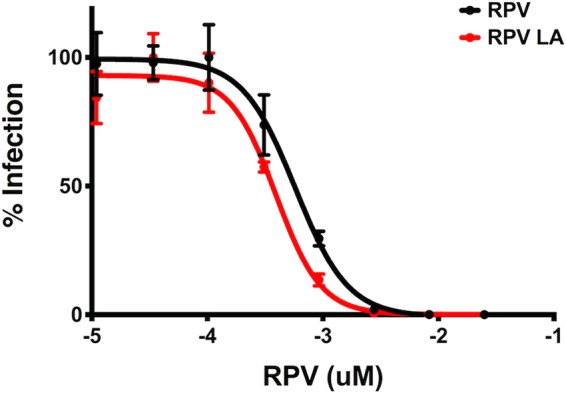
Inhibition of WT HIV-1_NL4-BAL_ by RPV and RPV LA *in vitro*. Infection of WT HIV-1_NL4-BAL_ was measured in TZM-bl cells in the presence of multiple concentrations of RPV or RPV LA. Error bars represent SD.

The RPV LA formulation that has been investigated in clinical trials was obtained from Janssen R&D Ireland. WT HIV-1_NL4-BAL_ was tested for RPV LA susceptibility in TZM-bl cells compared to that with soluble RPV (Fig. 2). The sensitivity curves for both drug formulations were similar, having EC_50_ values of 0.4 nM (0.15 ng/ml) and 0.6 nM (0.22 ng/ml) for RPV LA and RPV, respectively.

### RPV LA plasma and female genital tract tissue pharmacokinetics after a single dose.

**FIG 3:**
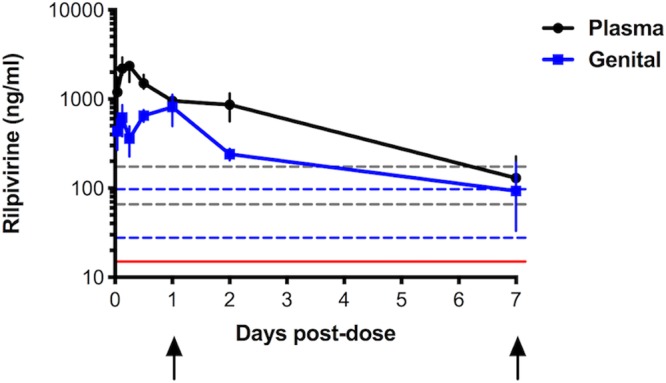
Plasma and female genital tract tissue RPV concentrations in female BLT mice after a single RPV LA dose. A single 150-mg/kg dose of RPV LA was administered to 14 female BLT mice. RPV concentrations were measured in plasma and female genital tract at 1, 3, 6, 12, 24, and 48 h and 7 days postdose. Each point represents the means from ≥2 animals, and error bars represent 95% confidence intervals. The dashed lines represent the range of measured concentrations from plasma (gray) or genital tract tissues (blue) from women during 28 days postdose (13). The red line indicates the previously reported protein-adjusted EC_90_ (12 ng/ml or 33 nM) (15). The arrows denote time points at which mice were challenged after RPV LA dose.

To assess the pharmacokinetics (PK) of RPV LA in humanized mice, 14 female BLT mice were injected intramuscularly with 150 mg/kg RPV LA. RPV concentrations were measured in the plasma and the female genital tract between 1 h and 7 days postinjection. The plasma RPV concentration peaked at 6 h, while the genital tract tissue concentration peaked at 24 h (Fig. 3). At 7 days postdose, the mean mouse plasma RPV concentration was 130 ng/ml, which was consistent with the concentration range reported within 28 days in women who received a single 1,200-mg RPV LA dose in multiple studies (13, 14). At 7 days postdose, the mean mouse genital tract tissue concentration was 93 ng/ml, which was approximately 2-fold higher than the mean concentration observed in vaginal tissue of women who received a 600-mg RPV LA dose (13). All mean RPV concentrations measured in the animals 7 days postdosing were 2- to 19-fold higher than the protein-adjusted EC_90_, 12.1 ng/ml, reported by Jackson et al. (13). Thus, biologically relevant plasma and female genital tract tissue concentrations of RPV were achieved within 7 days postdosing in this mouse model.

### Inhibition of WT HIV-1_NL4-BAL_ vaginal transmission by RPV LA PrEP.

**FIG 4:**
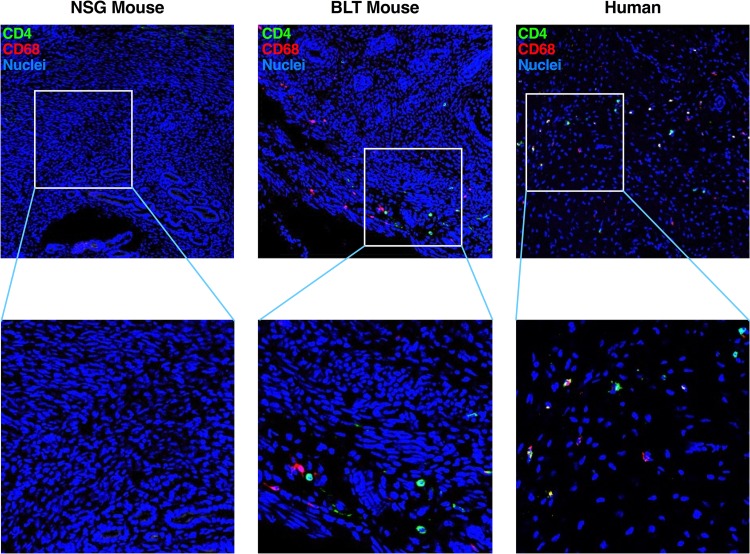
Immunofluorescent staining of human CD4 and CD68 in murine and human female genital tract tissues. Tissues were obtained and imaged from the female genital tract of a NSG mouse prior to reconstitution with human cells, a reconstituted BLT mouse, and cervical tissue from a human woman. Tissues were fixed, sectioned, and stained with anti-human CD4 (green) and anti-human CD68 (red) antibodies and Hoechst stain (nuclei; blue). Representative images are shown.

The focus of this study was to investigate the inhibition of vaginal transmission of WT HIV-1_NL4-BAL_ or RPV-resistant mutants in the presence or absence of PrEP. CD4^+^ and CD68^+^ cells were detected in the genital tract of BLT mice and in cervical tissue obtained from a healthy woman, but they were not detected in mice that were not reconstituted with human cells and tissues (Fig. 4). WT HIV-1_NL4-BAL_ and the Y181C and Y181V mutants were used for the PrEP study, and vaginal challenges were performed with 1 × 10^5^ infectious units (IU), as resolved by determining titers on GHOST cells, which was the minimum amount of virus to ensure that the majority of untreated control mice became infected. A quantitative reverse transcription-PCR (qRT-PCR) assay with single-copy sensitivity (single-copy assay; SCA) (51) was performed on plasma obtained for detection of viremia. Y181I HIV-1_NL4-BAL_ did not lead to detectable viral RNA in the plasma, suggesting that it conferred a significant fitness cost to the virus, and it was not further characterized *in vivo* (data not shown).

**FIG 5:**
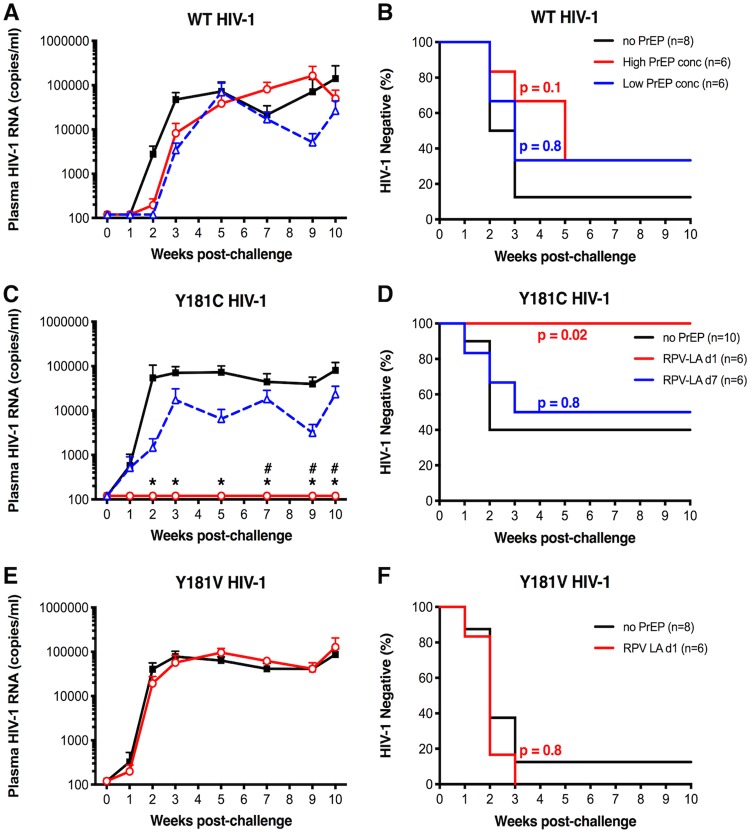
Plasma viremia and time to infection of RPV LA-treated animals after vaginal HIV-1_NL4-BAL_ challenge. (A, C, and E) Average plasma viral RNA levels in female BLT mice challenged vaginally with WT (A), Y181C (C), or Y181V HIV-1_NL4-BAL_ (E) after no treatment (black) or at day 1 (red) or 7 (blue) after RPV LA dose. Error bars represent the standard errors of the means (SEM). The limit of quantitation was 120 viral RNA copies/ml. *P* values of <0.05 are shown for time points comparing untreated versus RPV LA d1 groups (*) or untreated versus RPV LA d7 groups (#). (B, D, and F) Kaplan-Meier curves are shown for animals that remained negative over time after challenge with WT (B), Y181C (D), or Y181V (F) HIV-1_NL4-BAL_. *P* values between untreated and RPV LA treatment groups are shown.

To test whether biologically relevant plasma and tissue RPV concentrations could inhibit HIV-1, female mice were left untreated or received a single 150-mg/kg of body weight dose of RPV LA and then were challenged vaginally with WT or Y181C HIV-1_NL4-BAL_ 7 days later. HIV-1_NL4-BAL_ RNA was measured in plasma collected weekly for the first 3 weeks and bimonthly through week 10. Infection was defined as detectable plasma viral RNA from at least 2 time points. In the untreated group challenged with WT HIV-1_NL4-BAL_, 7 of 8 mice (88%) had detectable plasma viremia by 3 weeks postchallenge and remained positive through the last time point (Fig. 5A and B). Surprisingly, 4 of 6 animals (66%) challenged 7 days after RPV LA PrEP became infected at 2 to 3 weeks postchallenge. Additionally, one animal had detectable plasma HIV-1_NL4-BAL_ RNA at a single time point at week 7 postchallenge but remained negative thereafter. Spleen and genital tract tissues obtained from this animal at week 10 had undetectable viral RNA, suggesting that this animal remained uninfected (Table 1).

**TABLE 1:** HIV-1 *gag* RNA copies detected by SCA in plasma, spleen, and genital tract of untreated or PrEP-treated mice challenged with WT or Y181C HIV-1

Animal	Challenge HIV-1	Treatment	Plasma HIV-1 RNA (no. of copies/ml)	Spleen HIV-1 RNA (no. of copies/10^6^ CD4 RNA copies)^*a*^	Genital tract HIV-1 RNA (no. of copies/10^6^ CD4 RNA copies)^*b*^
912-012^*c*^	WT	RPV LA (d7)	<120	<1	<1
912-020^*c*^	Y181C	RPV LA (d7)	4,010	<1	<1
638-010^*c*^	WT	TFV/FTC	847	<1	<1
255-040^*d*^	WT	None	<120	<1	<1
255-005^*d*^	Y181C	RPV LA (d1)	<120	<1	<1
255-022^*e*^	WT	RPV LA (d1)	137,393	115,864	136,801,166^*f*^
638-003^*e*^	Y181C	None	276,573	20,649	498,570,981

a Samples had >9 × 10^4^ CD4 RNA copies.

b With the exception of animal 255-022, genital tract tissues had a range of 1.1 × 10^3^ to 2.6 × 10^6^ CD4 RNA copies.

c Animals had detectable plasma HIV-1 RNA at one time point.

d Animals served as negative controls in which all 7 plasma samples had undetectable HIV-1 RNA.

e Animals served as positive controls in which at least 5 plasma samples had detectable HIV-1 RNA.

f Genital tract tissue sample for this animal had 15 copies of CD4 and 2.1 × 10^3^ copies of HIV-1 *gag*.

As a previous study in humanized mice showed that long-acting RPV protected mice from vaginal challenge with WT HIV-1 at high, sustained plasma drug concentrations (52), a third group of mice was challenged with WT HIV-1_NL4-BAL_ at 1 day after RPV LA administration, at which time the average RPV concentrations were 7- and 9-fold higher in the plasma and genital tract, respectively, and remained well above the protein-adjusted EC_90_ for at least 6 additional days (Fig. 3). In this group, 4 of 6 mice (66%) ultimately became infected with WT HIV-1_NL4-BAL_, but 2/4 infected animals had delayed detectable plasma viremia until 5 weeks postchallenge (Fig. 5B). The differences in average viremia levels or time to infection in mice challenged with WT HIV-1_NL4-BAL_ in the presence or absence of RPV LA PrEP (day 1 [d1] or d7 postdose) were not statistically significant.

### Inhibition of RPV-resistant HIV-1_NL4-BAL_ vaginal transmission by RPV LA PrEP.

To determine if RPV LA could prevent vaginal transmission of RPV-resistant HIV-1 mutants, mice were challenged with the HIV-1_NL4-BAL_ molecular clone having either the Y181C RT mutation, which conferred 3-fold RPV resistance, or the Y181V RT mutation, which conferred 27-fold RPV resistance (Fig. 1A). Y181C HIV-1_NL4-BAL_ infected only 6 of 10 (60%) untreated mice, suggesting that the transmission and/or *in vivo* replication of this mutant was not as robust as that of WT HIV-1_NL4-BAL_ at the same infectious dose (Fig. 5C and D). While 3 of 6 (50%) mice challenged 7 days post-RPV LA dose became infected with Y181C HIV-1_NL4-BAL_ (*P* > 0.05), none of the mice challenged at 1 day after RPV LA PrEP became infected (*P* = 0.02). One animal challenged at day 7 after RPV administration was euthanized at week 7 postchallenge due to poor health (912-020) and had detectable plasma viremia at that last time point. HIV-1 RNA was not detected in spleen and genital tract tissues also taken at week 7 (Table 1), suggesting that this animal remained uninfected but had a false positive or could have had too few HIV-infected cells in the tissues at this time point to be detected. The average plasma viremia of mice infected with Y181C HIV-1_NL4-BAL_ during RPV LA was lower than that of untreated mice, which was statistically significant at 7 to 10 weeks postinfection (Fig. 5C).

Y181V HIV-1_NL4-BAL_ has approximately 9-fold greater resistance to RPV than Y181C HIV-1_NL4-BAL_. As two nucleotide changes are required to make Y181V compared to one nucleotide change to make Y181C, it is significantly less prevalent than Y181C *in vivo* (49). Despite similar replication levels *in vitro*, this virus was vaginally transmitted more easily than Y181C HIV-1_NL4-BAL_, with 7 of 8 (88%) untreated mice having detectable plasma viral RNA by week 2 or 3. This virus also was not inhibited by RPV LA PrEP. All mice (6/6) became infected when challenged with Y181V HIV-1_NL4-BAL_ 1 day after RPV LA administration (Fig. 5E and F). The plasma viremia curves of untreated and RPV LA-treated mice were indistinguishable, and the Kaplan-Meier curves showed no statistical difference.

**FIG 6:**
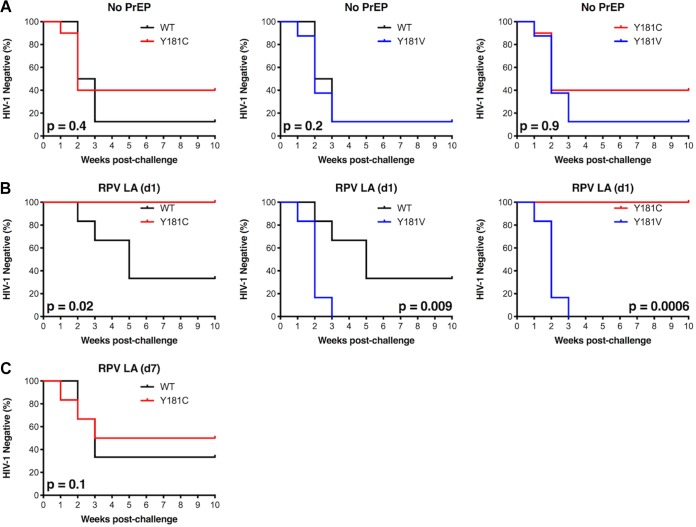
Comparison of time to infection of animals challenged with WT and mutant HIV-1_NL4-BAL_ either in the absence or in the presence of RPV LA PrEP. Kaplan-Meier curves are shown for all animals that remained negative over time in the absence of treatment (A), 1 day after RPV LA dose (B), or 7 days after RPV dose (C). *P* values between groups challenged with WT or mutant HIV-1_NL4-BAL_ are shown.

Kaplan-Meier curves were analyzed for WT, Y181C, and Y181V HIV-1_NL4-BAL_ in the absence or presence of RPV LA treatment to determine if vaginal transmission rates of viruses in the absence of RPV LA could account for differences in infection after RPV LA PrEP. Despite the fact that Y181C HIV-1_NL4-BAL_ was transmitted less efficiently than WT HIV-1_NL4-BAL_ or Y181V HIV-1_NL4-BAL_, there was no significant difference between the viruses in the time to transmission in untreated mice (Fig. 6A). In contrast, the transmission of WT HIV-1_NL4-BAL_ was significantly different from that of either of the 181 mutants when challenged 1 day after RPV LA (Fig. 6B). Specifically, in the animals challenged 1 day after PrEP, WT HIV-1_NL4-BAL_ infected some mice but Y181C HIV-1_NL4-BAL_ infected none of the mice, suggesting that reduced transmissibility of the Y181C mutant made it easier to be inhibited by RPV LA despite having low-level RPV resistance. While transmission of WT virus was sometimes inhibited by RPV LA PrEP (d1), Y181V HIV-1_NL4-BAL_ was never inhibited. Consistent with the lack of inhibition of WT HIV-1_NL4-BAL_ and Y181C HIV-1_NL4-BAL_ at d7 post-RPV LA treatment, time to infection did not differ between these two animal groups (Fig. 6C). These combined results show that high concentrations of RPV LA inhibited WT or Y181C HIV-1_NL4-BAL_ transmission more efficiently than Y181V HIV-1_NL4-BAL_.

### Inhibition of WT HIV-1_NL4-BAL_ vaginal transmission by TFV/FTC PrEP.

**FIG 7:**
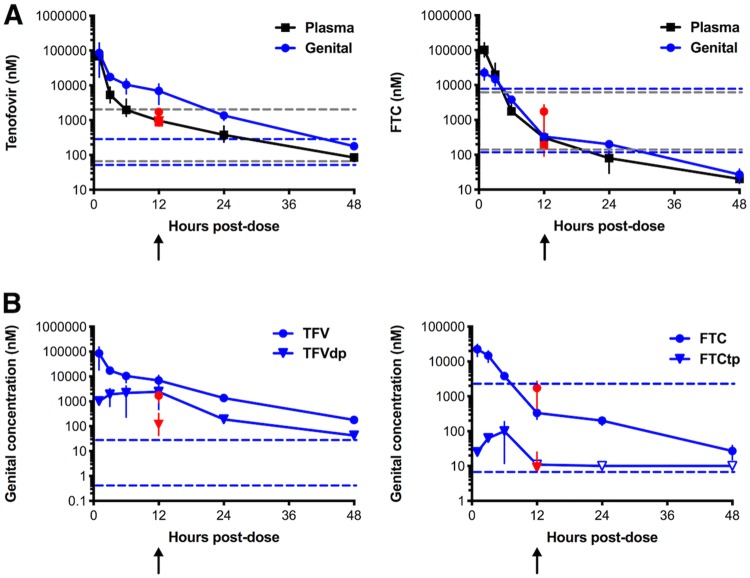
Plasma and female genital tract tissue drug concentrations in BLT mice after a single or multiple doses of TFV and FTC. A single dose of TFV (150 mg/kg) and FTC (100 mg/kg) was administered to 12 female BLT mice for PK measurements. (A) TFV (left) and FTC (right) concentrations were measured in plasma (black) and female genital tract (blue) at 1, 3, 6, 12, 24, and 48 h postdose. The dashed lines represent the range of measured TFV and FTC concentrations from human plasma (gray) or genital tract tissues (blue) (54). (B) TFV and FTC (blue circles) and active, phosphorylated TFV and FTC, TFVdp and FTCtp, respectively (blue triangles), were measured in the female genital tract. The blue dashed lines represent the range of measured TFVdp and FTCtp concentrations from human genital tract tissues (54). In addition, two doses of TFV (150 mg/kg) and FTC (275 mg/kg) were administered 24 h apart to an additional 3 animals, and the drugs and metabolites were measured 12 h after the last dose (red symbols). Each point represents the means from ≥2 animals, and error bars represent the interquartile ranges. The arrows denote the time point at which mice were challenged after TFV/FTC dose.

To determine if the WT HIV-1_NL4-BAL_ clone used in this study could be inhibited by Truvada PrEP, another group of mice was challenged vaginally during TFV/FTC treatment, similar to a study performed by Denton et al. (53). As previous studies had not shown both plasma and female genital tract tissue concentrations of TFV and FTC in BLT mice, we performed a PK study in which twelve female mice were administered a single dose of 150 mg/kg TFV and 275 mg/kg FTC. Drugs were measured in the plasma (unphosphorylated) and female genital tract tissue (both phosphorylated and unphosphorylated) between 1 and 48 h postdose. TFV concentrations in the plasma between 12 and 24 h were in the range of what has been observed in humans within 24 h of an oral Truvada dose (Fig. 7A, left), while the genital tract tissue concentrations of TFV and TFV diphosphate (TFVdp) (Fig. 7B, left) were approximately 10-fold higher than what has been observed in women (54).

FTC concentrations in the plasma and genital tract (Fig. 7A, right) were at the lower end of the ranges observed in women (54), and tissue FTC triphosphate (FTCtp) concentrations were undetectable at 12 h, 24 h, and 48 h postdose (Fig. 7B, right). Because the FTC/FTCtp concentrations were low at this dose, we increased the dose of FTC to 275 mg/kg in the virus challenge groups for TFV/FTC PrEP, keeping the TFV dose the same. The drug concentrations were similar to what we observed in the PK study and within the range of those observed in humans (Fig. 7A and B, right, red data points).

**FIG 8:**
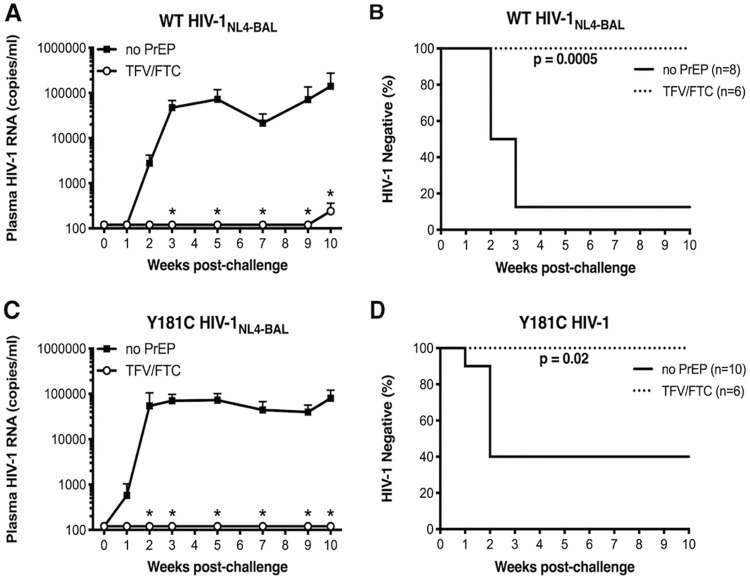
Plasma viremia and time to infection of TFV/FTC-treated animals after vaginal HIV-1_NL4-BAL_ challenge. (A and C) Average plasma viral RNA levels in female BLT mice challenged vaginally with WT (A) or Y181C (C) HIV-1_NL4-BAL_ after no treatment or during 5 days of TFV/FTC treatment. Error bars represent SEM. The limit of quantitation was 120 HIV-1 RNA copies/ml. *P* values of <0.05 are shown for time points comparing untreated to TFV/FTC-treated groups (*). (B and D) Kaplan-Meier curves are shown for animals that remained negative over time after challenge with WT (B) or Y181C (D) HIV-1_NL4-BAL_. *P* values are shown between treatment groups.

The mice were challenged with WT HIV-1_NL4-BAL_ or Y181C HIV-1_NL4-BAL_ 12 h after the first dose, and the animals were dosed daily for an additional 4 days. WT HIV-1_NL4-BAL_ was inhibited in 6 of 6 animals (100%) after vaginal challenge after TFV/FTC PrEP (*P* = 0.0005) (Fig. 8A and B). One of the TFV/FTC PrEP-treated animals (638-010) had detectable plasma viremia at week 10, which was the last time point collected, and did not meet our infection criteria that at least 2 positive plasma samples be detected. HIV-1 RNA was not detected in spleen and genital tract tissues taken at week 10, which could be due to either a false-positive result or to too few HIV-infected cells being detected in the tissues at the time of euthanasia (Table 1). Y181C HIV-1_NL4-BAL_ was inhibited by TFV/FTC PrEP in all animals (*P* = 0.02) (Fig. 8C and D).

### Inhibition of WT and RPV-resistant transmitted/founder HIV-1_CH185_ vaginal transmission by RPV LA PrEP.

**FIG 9:**
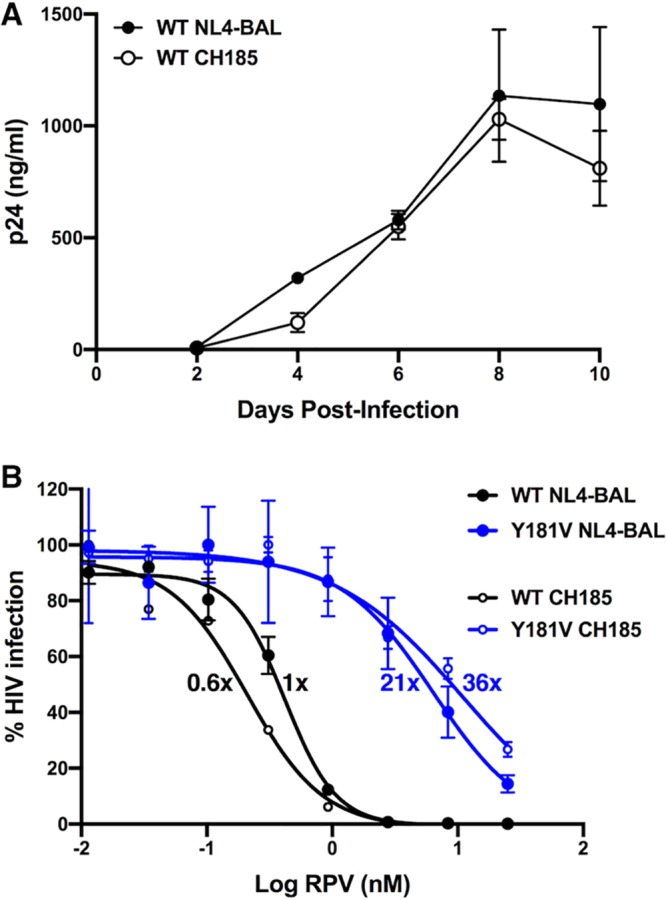
*In vitro* HIV-1_NL4-BAL_ replication and susceptibility to RPV. (A) Replication of WT HIV-1_NL4-BAL_ and WT HIV-1_CH185_ was measured in human primary PBMCs from 3 individual donors by production of p24 in cell culture supernatants. Results show means with SEM. (B) RPV susceptibility of WT and Y181V HIV-1_NL4-BAL_ as well as WT and Y181V HIV-1_CH185_ was measured in TZM-bl cells in the presence of multiple concentrations of RPV. The results are representative of 2 independent experiments, each performed in triplicate. Error bars represent SD. Average fold changes in resistance for each of the mutants compared to levels for WT HIV-1_NL4-BAL_ are designated next to each curve.

Surprisingly, WT HIV-1_NL4-BAL_, a laboratory-adapted molecular clone, was not completely inhibited by RPV LA even at high concentrations (d1), yet Y181C HIV-1_NL4-BAL_, which differed from WT virus by one nucleotide, was inhibited despite conferring 3-fold RPV resistance. As Y181C HIV-1_NL4-BAL_ was transmitted less efficiently *in vivo*, it is possible that the level of virus replication influences the ability of RPV LA PrEP to inhibit vaginal transmission. Thus, a biologically relevant virus, a molecular clone of a CCR5-tropic subtype C transmitted/founder (T/F) virus isolated from a woman, HIV-1_CH185_ (55), was evaluated *in vitro* and *in vivo*. The RT sequence of HIV-1_CH185_ had approximately 92% amino acid sequence identity to RT of HIV-1_NL4-BAL_. WT HIV-1_CH185_ and WT HIV-1_NL4-BAL_ replicated similarly in human PBMCs *in vitro* (Fig. 9A). Interestingly, HIV-1_CH185_ has the E138A polymorphism that is commonly observed in subtype C viruses (56), which has been shown to confer 2- to 3-fold resistance to RPV in laboratory-adapted viruses (57, 58). However, comparison of WT HIV-1_NL4-BAL_ and HIV-1_CH185_ showed that they have similar susceptibility to RPV *in vitro* (Fig. 9B). The average EC_50_ values were 0.34 ± 0.07 nM and 0.21 ± 0.003 nM, respectively. PCR mutagenesis was used to introduce Y181V in the HIV-1_CH185_ plasmid. RPV susceptibility assays comparing Y181V HIV-1_NL4-BAL_ with Y181V HIV-1_CH185_ gave average EC_50_ values of 7.3 ± 1.3 nM and 12 ± 1.5 nM, respectively, corresponding to 21- and 36-fold changes from the level for WT HIV-1_NL4-BAL_ (Fig. 9B).

**FIG 10:**
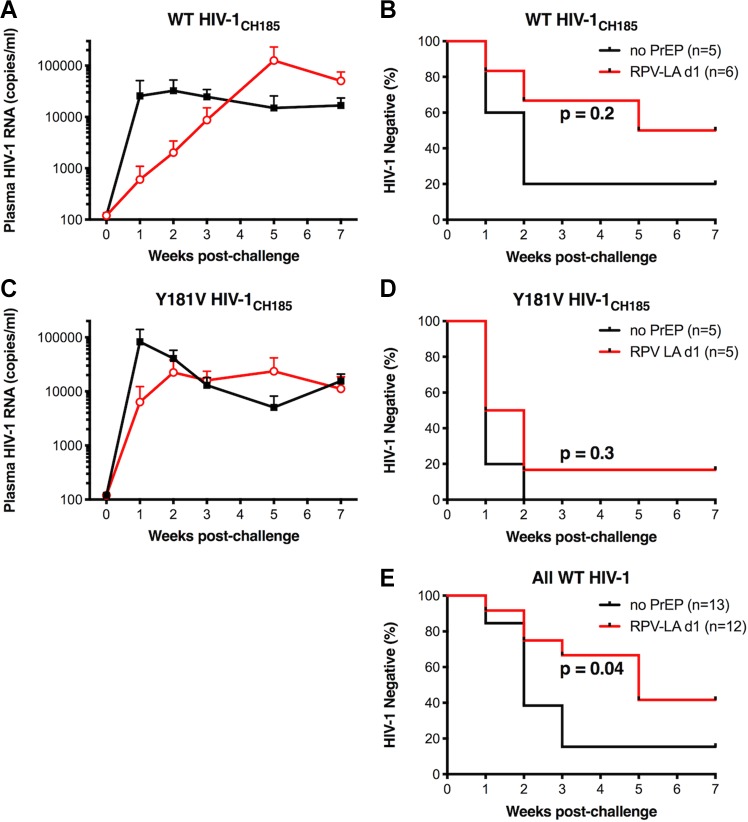
Plasma viremia and time to infection of RPV LA-treated animals after vaginal HIV-1_CH185_ challenge. (A and C) Average plasma viral RNA levels in female BLT mice challenged vaginally with WT (A) or Y181V (C) HIV-1_CH185_ after no treatment (black) or at day 1 after RPV LA dose (red). Error bars represent the SEM. The limit of quantitation was 120 viral RNA copies/ml. (B and D) Kaplan-Meier curves are shown for animals that remained negative over time after challenge with WT (B) or Y181V (D) HIV-1_CH185_. (E) Kaplan-Meier curves are shown for animals that remained negative after challenge with WT HIV-1_NL4-BAL_ or WT HIV-1_CH185_. *P* values are shown between untreated and RPV LA treatment groups. NS denotes a *P* value of >0.05 (not significant).

BLT mice were challenged vaginally with WT HIV-1_CH185_ or Y181V HIV-1_CH185_ in the absence of treatment or 1 day after RPV LA dosing. Similar to WT HIV-1_NL4-BAL_, WT HIV-1_CH185_ was transmitted in 80% of untreated mice by week 2 postchallenge (Fig. 10A and B). Initially, transmission of WT HIV-1_CH185_ was inhibited in 67% (4/6) of the mice after RPV LA prophylaxis through week 3, but a third animal had delayed plasma viremia that became detectable at 5 weeks postchallenge (Fig. 10A and B). The average plasma viremia level of mice infected with WT HIV-1_CH185_ after RPV LA treatment was lower than that of untreated mice, although this was not statistically significant (Fig. 10). In contrast, all of the animals challenged with Y181V HIV-1_CH185_ became infected, regardless of RPV LA PrEP (Fig. 10C and D). Although the Kaplan-Meier curves comparing transmission of WT and Y181V HIV-1_CH185_ show differences, this was not statistically significant, likely due to insufficient power. However, when all animals challenged with WT virus (NL4-BAL and CH185) are compared for untreated versus RPV LA (d1) treatment (Fig. 10E) or compared to levels for all Y181V-treated animals (data not shown), RPV LA does significantly inhibit transmission of WT HIV-1 and not Y181V HIV-1.

### New NNRTI-associated resistance mutations were detected in some mice at low frequencies in HIV-1_NL4-BAL_ RNA after infection during RPV LA PrEP.

A participant of the SSAT 040 clinical trial became infected with HIV-1 after receiving RPV LA PrEP and developed the RPV-associated resistance mutation K101E in plasma viral RNA (59). To determine if additional drug resistance mutations became detectable in mice that became infected during RPV LA PrEP, single-genome sequencing (SGS) of RT was performed on plasma viral RNA obtained from the last time point of 16/17 animals infected with WT, Y181C, or Y181V HIV-1_NL4-BAL_ during RPV LA PrEP. Five untreated control animals were included for comparison. Two of five untreated control mice had detectable new mutations associated with NNRTI treatment (Table 2), including one animal that had acquired H221Y at 2% frequency (Table 3). This mutation is associated with NNRTI treatment but does not confer resistance to NNRTIs, including RPV (60). Only one untreated control animal had detectable new RPV resistance mutations, which were present in nearly 10% of HIV-1 RNA genomes in this animal (Tables 2 and 3). Specifically, virus from that animal had acquired E138K, which confers 2-fold resistance to RPV (data not shown). After 10 weeks postinfection, transmitted Y181C or Y181V HIV-1 was still detected at 98 to 100% frequency in 4/4 animals (Table 3).

**TABLE 2:** Number of mice with new RT mutations detected in plasma HIV-1_NL4-BAL_ RNA by SGS at the last time point

Group	Mice (no./total no.) with:			
	New NNRTI-associated mutations	New RPV-resistant mutations	New RPV-resistant mutations of >5% frequency	M184I
WT untreated	0/1	0/1	0/1	0/1
Y181C untreated	1/2	0/2	0/2	0/2
Y181V untreated	1/2	1/2	1/2	0/2
All untreated	2/5	1/5	1/5	0/5
WT RPV (d1)	2/4	1/4	1/4	0/4
WT RPV (d7)	1/3	1/3	0/3	0/3
Y181C RPV (d7)	0/3	0/3	0/3	1/3
Y181V RPV (d1)	5/6	2/6	0/6	0/6
All RPV treated	8/16	4/16	1/16	1/16

**TABLE 3:** Frequencies of NNRTI-associated mutations detected in plasma HIV-1_NL4-BAL_ RNA at the last time point

Challenge virus and animal	Treatment (day of challenge)	NNRTI-associated mutation(s) (frequency)^*a*^
WT		
448-004	None	None (0/42)^*b*^
255-002	RPV LA (1)	None (0/43)
255-007	RPV LA (1)	None (0/44)
255-022	RPV LA (1)	H221Y (1/47)
255-044	RPV LA (1)	**K101E/E138K (1/47), E138K (3/47),** V179I (1/47)
912-008	RPV LA (7)	None (0/40)^*b*^
912-014	RPV LA (7)	None (0/44)^*b*^
943-004	RPV LA (7)	**E138Q (1/46)**^*b*^
255-042	RPV LA (7)	ND
Y181C		
448-024	None	C181Y (1/46),^*b*^^,^^*c*^ H221Y (1/46)
912-007	None	None (0/42)^*b*^
912-009	RPV LA (7)	None (0/44)^*b*^
912-017	RPV LA (7)	C181Y (1/46),^*b*^^,^^*c*^ M184I (2/46)
943-003	RPV LA (7)	None (0/46)^*b*^
Y181V		
255-031	None	**E138K (4/42)**
943-021	None	None (0/47)
255-006	RPV LA (1)	Y188H (1/45)
255-013	RPV LA (1)	V179I (2/43)
255-025	RPV LA (1)	V179I (3/46)
255-038	RPV LA (1)	**E138K (1/44),** P225H (1/44)
255-045	RPV LA (1)	**E138K (2/44),** V179I (1/44)
255-046	RPV LA (1)	None (0/46)

a NNRTI-associated mutations not detected in any mice were V90I, A98G, L100I, K101P/H/Q/R, K103X, V106X, V101I, I132X, E138A/G/R, V179D/E/F/L/T, and Y181I and any additional Y181C/V, Y188L/C, G190X, F227X, and M230I/L mutations. Boldface, RPV-resistant mutations. ND, not done.

b No mutations detected by population sequencing.

c Reversion to WT, not linked to additional resistance mutations.

Half of the infected RPV LA-treated animals (8/16) had new NNRTI-associated mutations, the majority of which were detected at low frequencies and in mice infected with Y181V HIV-1_NL4-BAL_ (Tables 2 and 3). Half of these animals (4/8) had only low-frequency mutations associated with NNRTI treatment that do not confer resistance alone, such as the mutations V179I (61, 62), Y188H (63, 64), H221Y (60), and P225H (63, 65). However, these mutations may enhance RPV resistance when present with substitutions at amino acid 181. The remainder of the mice with detectable NNRTI-associated RT mutations (4/8) acquired new mutations associated with RPV resistance, and all but one had resistance detected in <5% of viral genomes. These included E138K and E138Q, which confer low-level RPV resistance (49, 66). Population sequencing of viral RNA from a subset of these animals did not detect any of the mutations identified by SGS (Table 3). The one animal with acquired resistance at >5% frequency was infected with WT HIV-1_NL4-BAL_ at 1d post-RPV LA dose and had 1/47 genomes with K101E + E138K, 3/47 genomes with E138K alone, and 1/47 genomes with V179I. One mouse had M184I, which confers resistance to FTC, in 2/46 genomes despite not having been treated with TFV/FTC. All of the animals infected with viruses encoding Y181C or Y181V still had these mutations in 98 to 100% of their virus genomes.

**FIG 11:**
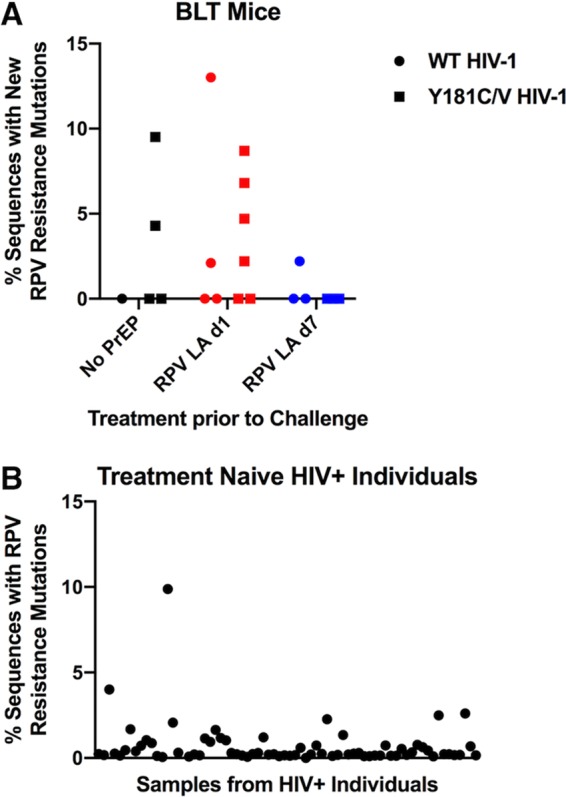
Percentage of HIV-1 sequences with RPV resistance in HIV-infected BLT mice and untreated humans. The frequencies of new RPV resistance mutations identified by SGS in HIV-infected mice in the untreated (black), RPV LA d1-treated (red), and RPV LA d7-treated (blue) groups (A) and RPV resistance mutations identified in a cohort of untreated HIV-infected people (67) (B) were plotted.

The percentage of sequences with RPV resistance mutations for the 12 mice in the RPV LA d1 group, the 6 mice in the RPV LA d7 group, and the 5 untreated mice were compared: 7/12 mice from the RPV LA d1 group, 1/6 mice from the RPV LA d7 group, and 2/5 mice from the untreated group had at least one sequence with RPV resistance (Fig. 11A). We found that the RPV LA d1 mice had significantly more resistance than the RPV LA d7 mice (*P* = 0.04), likely due to longer selection pressure by the drug. None of the other comparisons were significantly different (RPV LA d1 mice versus all other mice, RPV LA d7 mice versus no-PrEP mice, and combined RPV LA d1 mice and RPV LA d7 mice versus no-PrEP mice). To compare our results to those for humans, we determined the frequencies of RPV resistance mutations in HIV-1 from individuals who were treatment naive (Fig. 11B). MiSeq sequences were analyzed from 8 time points from 9 HIV-infected individuals who were treatment naive for a minimum of 5 years (67). The individuals were infected with subtype B (*n* = 7), C (*n* = 1), or 01_AE (*n* = 1) viruses, and each sample was treated individually. The highest percentage of sequences with an RPV resistance mutation for the human data set was 10%, which was similar to the range observed for the mouse data set. Although the average resistance frequency of the mouse data set was higher than that of the human data, this difference was not statistically significant (*P* = 0.8509).

## DISCUSSION

Preexisting NNRTI resistance has risen over the past decade, with current prevalence estimates ranging from 7 to 16% (46). Furthermore, the prevalence of preexisting NNRTI resistance in drug-naive individuals is twice as high in women (12%) as in men (6%) (46), suggesting that women are at greater risk of becoming infected with NNRTI-resistant viruses. These troubling statistics prompted us to study the ability of NNRTI-containing PrEP to prevent vaginal transmission of resistant HIV-1 mutants in a humanized mouse model.

All models have advantages and disadvantages. The humanized BLT mouse model leads to successful engraftment of lymphoid and mucosal tissues with human lymphocytes and myeloid cells, such that HIV-1 can be transmitted via multiple routes. However, the anatomy and functional adaptive immune responses in this model do not fully recapitulate humans or nonhuman primates. Nevertheless, this murine model was ideal to study the efficacy of the NNRTI RPV to prevent WT and drug-resistant HIV-1 infection via vaginal exposure. In addition, RPV does not inhibit simian immunodeficiency virus (SIV) and is rapidly metabolized in macaques even at a high dose (68), precluding the use of that animal model.

Administration of a single dose of RPV LA to female humanized mice led to similar plasma and genital tract RPV concentrations detected in human women, although they declined rapidly. Biologically relevant concentrations of RPV in the genital tract and plasma at day 7 were not sufficient to prevent vaginal transmission of a highly infectious HIV-1 molecular clone (NL4-BAL) with or without a common drug resistance mutation, Y181C. This was in contrast to daily TFV/FTC treatment 2 days prior to and 3 days after challenge with the same viruses. RPV, unlike nucleoside analogs, is highly protein bound, like other NNRTIs (25, 69), making it difficult to distinguish between active drug and protein-bound drug in tissues or plasma.

Surprisingly, even 8-fold higher concentrations of RPV present 1 day after RPV LA injection inhibited vaginal transmission of WT HIV-1 in some mice, often merely delaying detection of plasma viremia. These results suggest that the drug suppressed virus replication initially but that waning drug concentrations over time allowed virus to replicate. However, vaginal transmission of Y181C HIV-1 was inhibited in all animals despite having 3-fold resistance, suggesting that this level of resistance is not clinically relevant in the context of RPV LA PrEP at high enough concentrations. Y181C HIV-1 had slightly less transmissibility via the vaginal route than WT virus at the same *in vitro* infectious dose (60% versus 88%) in the absence of drug but similar replication *in vivo*. Thus, it is likely that increased initial *in vivo* replication can decrease the efficacy of RPV LA PrEP. A similar effect was observed in a macaque study in which animals were protected by TFV/FTC PrEP when rectally challenged with simian-human immunodeficiency virus with the M184V RT mutation, which confers high FTC resistance but significantly reduced replication fitness and modest hypersensitivity to TFV (70). The additional base substitution to change Y181C to Y181V increases RPV resistance 8-fold and rendered HIV-1 able to be transmitted in all animals in the presence of higher RPV PrEP concentrations.

NNRTIs noncompetitively inhibit HIV-1 reverse transcriptase after the virus enters the target cell. If not all RT molecules are inhibited by an NNRTI, the virus replication cycle can proceed. In our study, the majority of untreated animals that became infected (90%, 26/29) had detectable plasma HIV-1 RNA by 2 weeks after challenge regardless of virus used, with the remaining 10% becoming detectable at week 3 postchallenge. However, a delay in detectable plasma viremia (week 5) was observed in 3/7 animals that were challenged at high RPV concentrations with WT HIV-1 (255-002, 255-007, and 125-07), suggesting that RPV reduced virus replication, particularly as one animal had a noticeably lower viremia set point than the others. These results suggest that low frequencies of cells become infected with HIV-1 during PrEP, and virus replication and/or proliferation of HIV-infected cells occurred only when drug levels decreased. While further studies should be conducted to understand HIV-1 entry and infection of local target cells during PrEP treatment, our results indicate the importance of maintaining PrEP at sustained levels after virus exposure to prevent potential rare infection events in the mucosa from disseminating systemically.

Our study differs slightly from that of Kovarova et al., in which 7.5 to 15 mg of RPV LA was administered to BLT mice, leading to sustained plasma RPV concentrations of ≥400 to 700 ng/ml for 4 weeks and no WT HIV-1 (JR-CSF or subtype B T/F) transmission (52). Animals in our study were administered drug based on animal body weight rather than a fixed dose; on average, 3 mg RPV LA (2.5- to 5-fold less than the dose used by Kovarova et al.) was administered to our mice. This resulted in similar plasma and genital tract levels and decay slopes seen in human women, particularly at day 7, which was chosen for virus challenge (13). Even when mice were challenged with WT HIV-1 on day 1 after RPV LA dosing, in which the mice had plasma RPV concentrations similar to those observed by Kovarova et al., plasma RPV was not sustained at that level for several weeks in our study. This long-term release of RPV into the plasma in the other study is likely due to a relatively large bolus of the nanoparticles, as we saw similar effects when a human dose of RPV LA was delivered to macaques (68). The combined results from these two humanized mouse studies suggest that high plasma RPV concentrations should be sustained to prevent transmission of highly transmissible WT HIV-1. Clinical RPV LA PK studies have shown mixed results, such that plasma concentrations in women may be lower than those in men (13), and RPV LA concentrations in vaginal or endocervical tissues were insufficient to inhibit WT HIV-1 infection *ex vivo* (71, 72). Exposure to virus during RPV LA PrEP when drug concentrations are waning may result in infection. However, the current study suggests that highly resistant viruses with strong replication capacity cannot be inhibited even when high RPV concentrations are sustained. NNRTI-resistant clinical isolates with different mutations will be important to study in this model to understand their transmissibility and ability to be inhibited by PrEP agents already approved or under investigation.

Despite RPV-resistant HIV-1 transmission during RPV LA PrEP, detectable new RPV resistance mutations were seen in only 25% of the animals. This was not significantly different from the frequency of detectable resistance observed in a subset of untreated animals or in HIV-infected people. Only one animal in each of the untreated and RPV LA-treated groups had RPV-resistant mutations in more than 1 to 2 RNA genomes, which were only detected by SGS and not population-based sequencing. These results are consistent with our previous pilot macaque study in which RT-SHIV-infected macaques were treated with RPV LA and developed E138K/Q at ≤5% frequency in plasma viral RNA at a single time point (68). E138K was the most prevalent RPV resistance mutation detected in the current mouse study, including in approximately 10% of genomes isolated from an untreated control infected with Y181V HIV-1. Interestingly, E138K did not arise in the virus of animals infected with Y181C HIV-1_NL4-BAL_, which is likely due to this combination of mutations leading to a less fit virus (57).

In addition, M184I, a FTC resistance mutation, was detected in approximately 5% of genomes from a RPV LA-treated animal. Both E138K and M184I have been shown to develop as a result of APOBEC3-mediated G-to-A hypermutation of viral DNA (73–76), which may explain the presence of these HIV-1 mutants in animals not exposed to antiretroviral drugs that select them. V179I developed in HIV-1 from 4 RPV LA-treated animals, similar to several clinical studies that demonstrate that this site is highly polymorphic in NNRTI-treated individuals but does not confer NNRTI resistance (61, 62, 77, 78). Interestingly, V179I also arises as a result of a single G-to-A base change.

High-level RPV resistance arises in HIV-1 with two mutations that result in either a single amino acid substitution (e.g., K101P or Y181I/V) or multiple amino acid substitutions (e.g., L100I + K103N) in RT (66). In this study, classic RPV-associated resistance mutations (K101E, E138K, and E138Q) arose at low frequency in a subset of both RPV LA-treated animals and untreated animals. However, these mutations arise from a single base change and confer only 2- to 5-fold resistance to RPV, according to our data and those of others (25, 58, 60, 65, 68, 79). It is possible that some mutations selected in genomes already encoding Y181V lead to additive levels of resistance, but this is unlikely to have much effect in a virus that already has >20-fold reduced susceptibility to RPV and that can be transmitted despite high concentrations of drug in tissues and blood.

Overall, the selection of low-level drug-resistant HIV-1 at very low frequencies in a quarter of the animals infected during RPV LA treatment is encouraging. High-level RPV resistance in HIV-1 required by two or more mutations likely did not have time to develop in this 10-week mouse study or in our previous 30-week macaque study (68). This also could be the case for other PrEP agents, either singly or in combination, that require multiple mutations to acquire significant drug resistance. In fact, recent data reported for the phase III trial of vaginal rings containing dapivirine, an NNRTI related to RPV, showed that additional NNRTI-associated resistance mutations did not arise in a subset of women who seroconverted after using the ring (80). However, the impact of low-frequency RPV-resistant HIV-1 on virologic outcome during ART, particularly regimens containing NNRTIs, is not yet known.

## MATERIALS AND METHODS

### Viruses.

HIV-1_NL4-BAL_, HIV-1_CH185_, and avian sarcoma-leukosis virus (RCAS) were produced by transfection of HEK293T cells with a plasmid encoding the proviruses (81, 82), kindly provided by Ned Landau, Christina Ochsenbauer, and Stephen Hughes, respectively, with Lipofectamine 2000 (Invitrogen). The mutation Y181C was introduced into the HIV-1_NL4-BAL_ plasmid, and the mutation Y181V was introduced into the HIV-1_NL4-BAL_ and HIV-1_CH185_ plasmids by PCR mutagenesis using the QuikChange XL kit (Stratagene) or Q5 site-directed mutagenesis (New England Biolabs). HIV-1 infectivity was determined 48 h after limiting dilution on GHOST-R3/X4/R5 cells (83) by flow cytometry.

### Antiretroviral drugs.

RPV (number 12147) for *in vitro* assays was obtained through the NIH AIDS Reagent Program, Division of AIDS, NIAID, NIH, from Tibotec Pharmaceuticals, Inc., and stored at –20°C in dimethyl sulfoxide (DMSO). RPV LA (300 mg/ml) was obtained from Janssen R&D Ireland and stored at 4°C until use. TFV and FTC were obtained from Gilead Sciences.

### Drug susceptibility assay.

WT, Y181C, and Y181V HIV-1 stocks were phenotyped for resistance to RPV or RPV LA as previously described (68). Briefly, TZM-bl cells (84) were seeded at 5 × 10^3^ cells in 96-well cell culture-treated, white-walled plates (PerkinElmer) in phenol red-free Dulbecco’s modified Eagle’s medium (DMEM; Life Technologies) supplemented with 10% fetal bovine serum (FBS; Atlanta Biologicals) and 100 U/ml penicillin, 100 μg/ml streptomycin, and 0.292 mg/ml l-glutamine (PSG; Life Technologies). The following day, virus was added at a multiplicity of infection (MOI) of 0.05 in the presence or absence of RPV dilutions in triplicate. Plates were incubated at 37°C for 48 h, and luciferase was measured using Britelite Plus reagent (PerkinElmer) on a Luminoskan Ascent microplate luminometer (Thermo Scientiﬁc). Relative light units (RLU) were converted to percent infection by dividing the RLU of each drug dilution by the RLU of the 100% infection control in the absence of drug. Wells containing cells with no virus and no drug were used to normalize for background luciferase output. The EC_50_ was calculated by log transforming drug concentrations and using a four-parameter variable-slope nonlinear regression for curve-ﬁtting analysis using PRISM 6 (GraphPad).

### HIV-1 replication assay.

Replication of HIV-1 viruses was performed in PBMCs. PBMCs were isolated from blood obtained from HIV-1-uninfected donors at the Central Blood Bank and overlaid on lymphocyte separation medium (MP Biomedicals). Cells were stimulated for 48 h with 5 μg/ml phytohemagglutinin in RPMI containing 10% FBS, PSG, and 50 U/ml interleukin-2 (IL-2; Roche). Cells were washed and incubated with HIV-1 at an MOI of 0.01. Production of capsid (p24) antigen was measured by enzyme-linked immunosorbent assay (ELISA; Xpress Bio) in supernatants taken between 2 and 10 days postinfection after filtration through a 0.45-μm syringe filter.

### Ethics statement.

All animal-related work was conducted according to the Public Health Services (http://grants.nih.gov/grants/olaw/references/PHSPolicyLabAnimals.pdf). Mice were housed at the University of Pittsburgh Division of Laboratory Animal Resources in accordance with the American Association of Accreditation of Laboratory Animal Care standards. All procedures were approved by the University of Pittsburgh Institutional Animal Care and Use Committee under protocol 17020145. Animals were housed in microisolator cages and monitored daily for behavior, appearance, and physiology. Mice had unlimited water, which was autoclaved, acidified, and treated on alternate weeks with sulfamethoxazole-trimethoprim, and dry food. DietGel 76A cups (ClearH2O) often were provided in cages to supply extra nutrition postprocedure. All procedures were conducted while the animals were sedated either with an intramuscular injection of ketamine (44 mg/kg; Henry Schein) or inhalation of isoflurane (5% in 70% O_2_-30% NO_2_). Animals were euthanized at the endpoint of the study with 100% carbon dioxide at a flow rate of 20 to 30%. Any animal that failed to thrive (i.e., loss of 20% body weight from the start of the experiment), ambulate, or perform normal mouse behavior or was obviously moribund was also euthanized.

### Animals.

Female NSG (NOD.*Cg-Prkdc^scid^Il2rg^tm1Wjl^*/SzJ) mice were purchased from Jackson Laboratory. Reconstitution of 119 mice with human immune cells from 7 different donors (designated 125, 255, 270, 448, 638, 912, and 943) was performed by Jackson Laboratory or in our laboratory via transplantation of fetal human CD34^+^ cells, liver, and thymus (BLT) obtained from the University of Pittsburgh Biospecimen Core, an Institutional Review Board-approved Honest Broker System, as previously described (85). Human immune cell reconstitution was determined by flow cytometry on PBMCs isolated from the mice and stained with anti-mouse CD45-fluorescein isothiocyanate, anti-human CD45-phycoerythrin (Miltenyi), and anti-CD3-V450 antibodies for animals produced in our laboratory. Animals from The Jackson Laboratory or our laboratory were used only if they had >25% peripheral human CD45^+^ cells.

### *In vivo* pharmacokinetics of RPV LA and TFV/FTC.

For RPV LA, 14 mice were injected intramuscularly in the flank with 3 mg RPV LA, for a dose of approximately 150 mg/kg. Two mice were euthanized at 1, 3, 6, 12, 24, 48, and 168 h postinjection. For TFV/FTC, an additional 12 mice were injected intraperitoneally with 150 mg/kg TFV and 100 mg/kg FTC. Two mice were euthanized at 1, 3, 6, 12, 24, and 48 h postinjection. Blood was drawn at each time point until euthanasia, and plasma was separated. At necropsy, female genital tract and colon/rectum were collected, weighed, and immediately flash-frozen dry.

RPV, TFV, and FTC were extracted from mouse plasma with isotopically labeled internal standards (RPV-d_6_; TFV-^13^C_5_; and FTC-^13^C, ^15^N_2_, respectively) using protein precipitation. TFV and FTC were separated on a Waters Atlantis T3 (2.1 by 50 mm, 3 μm) high-performance liquid chromatography (HPLC) column and RPV on an Xterra MS C_18_ (2.1 by 50 mm, 3.5 μm) HPLC column. The calibrated ranges were 1 to 4,000 ng/ml (TFV and FTC) or 1 to 10,000 ng/ml (RPV). Calibration standards and quality control (QC) samples were prepared in blank plasma with an acceptance criteria of ±20%.

Frozen tissue biopsy specimens were homogenized in 1 ml of cold 70:30 acetonitrile–1 mM ammonium phosphate (pH 7.4). RPV, TFV, FTC, TFVdp, and FTCtp were extracted from tissue homogenate with the following isotopically labeled internal standards: RPV-d_6_; TFV-^13^C_5_; FTC-^13^C, ^15^N_2_; and TFVdp-^13^C_5_ (for both TFVdp and FTCtp). For quantification, analytes and internal standards were separated on a Waters Atlantis T-3 C_18_ HPLC column (TFV and FTC), a ThermoBioBasic AX HPLC column (TFVdp and FTCtp), or an Xterra MS C_18_ (2.1 by 50 mm, 3.5 μm) HPLC column (RPV). The calibrated ranges were 0.300 to 300 ng/ml (TFV, FTC, TFVdp, and FTCtp) and 1 to 200 ng/ml (RPV). Resulting concentrations were normalized to tissue weight assuming a tissue density of 1 g/ml (86) and reported as nanograms or femtomole per gram. Calibration standards and QC samples were prepared in blank tissue homogenate with an acceptance criteria of ±20%.

### Immunofluorescence staining.

Genital tract tissues from NSG mice with or without human fetal tissue engraftment, as well as human female cervical tissue (a kind gift from Charlene Dezzutti), were obtained and flash-frozen dry. Later, the tissues were thawed in 2% paraformaldehyde at 4°C and placed in 30% sucrose for 24 h. Tissues were immersed in 2-methylbutane cooled in liquid nitrogen for 30 s. Cryosections (≤9 μM) were placed on slides and stained with Hoechst, anti-CD4 antibodies (ab4055; Abcam), and anti-CD68 antibodies (clone EBM11; Dako) and imaged at 40× with a Nikon A1 spectral confocal microscope at the University of Pittsburgh Center for Biologic Imaging.

### *In vivo* HIV-1 challenge studies.

For virus challenge studies, an initial pilot study was performed on animals that were challenged atraumatically via the vaginal canal with 5 × 10^4^ IU of WT HIV-1_NL4-BAL_ or the Y181C or Y181I mutant. Animals that did not become infected after 4 weeks were rechallenged with 1 × 10^5^ IU. If they were still uninfected after an additional 4 weeks, they were rechallenged with 1.5 × 10^5^ IU. It was determined that a challenge dose of 1 × 10^5^ IU of WT HIV-1_NL4-BAL_ or Y181C HIV-1_NL4-BAL_ was necessary to infect the majority of untreated animals after one challenge.

The initial vaginal challenge of 3 untreated BLT mice with 1 × 10^5^ IU HIV-1_CH185_ did not result in infection of the mice. Therefore, 2 × 10^5^ IU was used for this virus to ensure that the majority of the untreated animals became infected.

Mice were left untreated or were given PrEP as (i) a single intramuscular injection of 3 mg RPV LA or (ii) 150 mg/kg TFV and 275 mg/kg FTC given daily 2 days before and 3 days after challenge. Mice were challenged intravaginally with 1 × 10^5^ IU of HIV-1_NL4-BAL_ (WT, Y181C, or Y181V) or 2 × 10^5^ IU of HIV-1_CH185_. All challenges were performed atraumatically to prevent tearing of the tissue. The volume of challenge virus was less than 15 μl, and animals remained anesthetized for at least 15 min under observation to ensure that the liquid was absorbed. There were 6 to 10 animals per group. Peripheral blood was drawn weekly or bimonthly in EDTA-containing tubes for 7 to 10 weeks after challenge. Plasma was separated and stored at –80°C. At 7 to 10 weeks, mice were euthanized and plasma and tissues were harvested and stored at –80°C or in liquid nitrogen, respectively.

### Viral RNA quantitation.

Plasma HIV-1 RNA was isolated as previously described (87) and quantified by SCA (51). Briefly, a known amount of RCAS virus was spiked into each 50-μl plasma sample as an internal RNA isolation control, and HIV-1 and RCAS were pelleted by centrifugation at 4°C. Total viral RNA was isolated with guanidinium isothiocyanate and glycogen, and cDNA synthesis was performed with random hexamers. TaqMan quantitative PCR was performed in duplicate for all samples and RNA standards prepared by *in vitro* transcription. HIV-1_NL4-BAL_ *gag* and RCAS RNA transcripts for standard dilutions were synthesized from plasmids encoding the region of amplification using the RiboMAX large-scale RNA production system (Promega). As the sequence of HIV-1_CH185_ differed from that of HIV-1_NL4-BAL_, the probe and forward primer were modified for samples from animals infected with this T/F virus, and a sequence-matched RNA standard was derived from a custom RNA oligonucleotide (IDT). Data were only reported from samples in which RCAS was successfully amplified. The limit of quantitation of SCA from the plasma volumes was 120 HIV-1 RNA copies/ml plasma.

Intracellular HIV-1 RNA from tissues was isolated as previously described (88). Briefly, tissues were homogenized using a TissueLyser (Qiagen) in the presence of lysis buffer (RTL; Qiagen) and 20 U of RNase inhibitor. RNA was extracted from lymphoid tissues, lung, liver, and genital tract tissues using the RNeasy kit (Qiagen) in a total of 50 μl RNase-free water and stored at –80°C. HIV-1 SCA was performed on RNA as described above, with the exception of the RCAS internal control. Human CD4 RNA copies or total RNA was used for normalization.

### Plasma HIV-1 sequencing.

Population sequencing was performed on vRNA isolated from 50 μl plasma obtained at the last time point from a subset of mice using nested PCR. cDNA was generated from vRNA using the SuperScript III first-strand synthesis system (Thermo Fisher Scientific), using random hexamer primers. The entire RT coding region was amplified using the primers BAL3F (5′-TGTGGAAAGGAAGGACACC-3′) and BAL5R (5′-TCACTATTATCTTGTATTACTACTGC-3′) with the following reaction conditions: 94°C for 2 min; 40 cycles of 94°C for 15 s, 49°C for 30 s, and 68°C for 3 min; and 1 cycle of 68°C for 5 min. The second round of PCR ampliﬁcation was performed using primers RT-F (5′-TTTGCCAGGAAGATGGAAAC-3′) and RT-R (5′-TCACTAGCCATTGCTCTCCA-3′) with the following reaction conditions: 94°C for 2 min; 26 cycles of 94°C for 15 s, 63°C for 30 s with −0.5°C increments per cycle, and 72°C for 2 min; 15 cycles of 94°C for 15 s, 52°C for 30 s, and 72°C for 2 min; and 1 cycle of 72°C for 5 min. The Platinum *Taq* DNA polymerase high-fidelity kit (Thermo Fisher Scientific) was used for both rounds of PCR. PCR products were purified with the Wizard SV gel and PCR clean-up system (Promega). RT was sequenced using primers RT-F, 200F (5′-GTAGGATCTGACTTAGAAA-3′), 350F (5′-CAGGAAAATATGCAAGAATG-3′), and RT-R.

SGS was performed by limiting dilution of cDNA, as previously described (89). Briefly, cDNA was diluted to a single copy, and an amplicon of the first 831 bp of RT was generated and sequenced. Forty to 47 sequences were obtained from each sample.

### Statistical analyses.

The Mann-Whitney Wilcoxon test was performed to determine differences in the distribution of plasma viremia levels between mouse treatment groups. The log rank Mantel-Cox test was performed on Kaplan-Meier curves to determine differences in time to infection between mouse groups. The one-sided Mann-Whitney U test was performed to compare the frequencies of plasma HIV-1 mutations detected by SGS in different mouse treatment groups and to analyze human data. A *P* value of <0.05 was considered statistically significant for all tests.
